# Parent-adolescent communication on sexual and reproductive health issues and its associated factors in Ethiopia: a systematic review and meta-analysis

**DOI:** 10.1186/s13052-020-00921-5

**Published:** 2020-10-30

**Authors:** Maru Mekie, Dagne Addisu, Abenezer Melkie, Wubet Taklual

**Affiliations:** 1 Department of Midwifery, College of Health Sciences, Debre Tabor University, Debre Tabor, Ethiopia; 2 Department of Public Health, College of Health Sciences, Debre Tabor University, Debre Tabor, Ethiopia

**Keywords:** Parent-adolescent communication, SRH, Prevalence, Systematic review, Meta-analysis

## Abstract

**Background:**

Every year, 1.3 million young people reported to die from preventable causes of death. Parent-adolescent communication on sexuality is critical in informing youth about risk and protective behaviors which in turn decrease the likelihood of involvement in risky sexual behaviors. This systematic review and meta-analysis was intended to assess the prevalence of parent-adolescent communication on sexual and reproductive health (SRH) issues and its associated factors in Ethiopia.

**Methods:**

PubMed, EMBASE, HINARI, Google Scholar, and University repositories were used to search studies. Article search was conducted from May 20 to June 9, 2020. Critical appraisal of studies was conducted using Newcastle-Ottawa Quality Assessment Scale (NOS). Data analysis was conducted using Stata 11 software following the abstraction of data using a format prepared on Microsoft excel. The heterogeneity of studies was tested using Cochran (Q test) and I^2^ test statistics. Similarly, funnel plot and Egger’s regression asymmetry were used to assess publication bias. Subgroup analysis was conducted based on study Regions and sample size.

**Result:**

Fourteen studies with sample of 8018 adolescents were included in this systematic review and meta-analysis. The pooled prevalence of parent-adolescent communication on SRH issues in Ethiopia was found to be 45.18% (95%, CI, 32.23, 58.13%). Adolescents’ knowledge of reproductive health matters (OR = 2.91, 95% CI:1.21, 7.01), believe on importance of discussion on SRH issues (OR = 4.18, 95% CI: 2.63, 6.65), had history of sexual exposure (OR = 1.95, 95% CI: 1.53, 2.50), parents openness to discuss SRH issues (OR = 3.39, 95% CI: (2.48, 4.62), and being female (OR = 1.60, 95% CI:1.07, 2.38) were the positive predictors of parent-adolescent communications on SRH issues.

**Conclusion:**

The prevalence of parent-adolescent communication on SRH issues was found to be low. Knowledge of adolescents about reproductive health matters, believe on the importance of discussion on reproductive health issues, history of sexual exposure, parents’ openness to discuss SRH issues, and being female were found to be the positive predictors of parent-adolescent communication on SRH issues in Ethiopia. The finding our study indicated that evidence based education about reproductive health matters could significant to improve adolescent parent communication on SRH issues.

## Introduction

Adolescent population accounts for a quarter of the global population and more than 70% resides in low income countries [1]. Time of adolescence is a key phase of human development. The rapid biological and psychosocial changes that take place during the second decade affect every aspect of adolescents’ lives. Period of adolescence is an important time for laying the foundations of good health in adulthood if appropriate and adolescent tailored health strategies are established. Biological maturity usually precedes psychosocial maturity in which most adolescents try to do experimentation what they have observed around [2, 3].

Adolescents are portion of population who are not served well by the existing health services. In order to have universal health coverage following the post 2015, it is important to ensure that the adolescent segment of the population receives adequate attention [3]. Many adolescents are reported to die prematurely due to accidents, suicide, violence, pregnancy related complications, and other reproductive illnesses that are either preventable or treatable through provision of adolescent tailored reproductive health services [2, 4]. According to WHO report, 1.3 million youth population reported to die every year from preventable causes of death [5]. Adolescent population are disproportionately affected by Human Immuno- Deficiency Virus (HIV)/ (Acquired Immuno-Deficiency Syndrome (AIDS), and other reproductive health problems. Eighty-two percent of the estimated 2.1 million adolescents aged 10–19 years living with HIV were in Sub-Saharan Africa (SSA) as of the 2012 report in which 58% of them were females. The review indicated that comprehensive knowledge about HIV, condom use, HIV testing, and antiretroviral treatment coverage remain low in most low income countries [6]. Globally, about 16 million women 15–19 years old give birth each year. Ninety-five percent of these births occur in low- and middle-income countries [2].

In Ethiopia, more than 60% of the population are under 24 years of age. On the other hand adolescent population (age 10–19 years) accounts the quarter of the total Ethiopian population which demands large investment in reproductive health [7]. Youth population who have limited access to quality youth friendly services are at increased risk of negative reproductive health outcomes. The Federal Ministry of Health of Ethiopia (FMoH) has established National adolescent and youth reproductive health strategy to improve the health of young population through training of health care providers at all levels, mobilizing resources to ensure continuous supplies of commodities, engagements partners, and communities to increase demand and knowledge of adolescents and youth reproductive health services [8]. However, adolescents and youth reproductive health has not been improved and reproductive health problems are continue to be a problem among young people in Ethiopia.

Parent-adolescent communication on sexuality is critical in informing young people about risk and protective behaviors which in turn decrease the likelihood of involvement in risky sexual behaviors [9, 10]. Parents are one of the primary stakeholders who can play important roles in protecting adolescents from risky sexual behaviors such as unsafe sex, unwanted pregnancy, substance use, and violence [2, 3, 11]. Parents who are open enough for their young child with regards to sexuality have better communication which is important to reduce risky sexual behaviors such as early sexual initiation, unwanted pregnancy, and other reproductive health problems [3, 12].

Different factors are found to affect adolescent parent communication on sexual and reproductive health (SRH) issues. Cultural taboo, feel embraced to discuss on sexual issues, lack of communication skill, belief on sexuality, and knowledge on sexuality were some of the factors which affect adolescent parent communication on SRH issues [12–14]. Effective parent-adolescent communication is important to reduce adolescents’ engagement in risky sexual behaviors. Many individual studies were conducted in different Regions of Ethiopia [12, 13, 15–17]. However, comprehensive evidence is lacking with regards to adolescent parent communication on SRH issues and its determinant factors. This systematic review and meta-analysis aimed to assess the pooled prevalence of adolescent parent communication and its determinant factors in Ethiopia.

## Methods

A systematic review and meta-analysis was conducted to estimate the prevalence of parent-adolescent communication on SRH issues and its determinant factors.

### Search strategy and study selection

Both published and unpublished studies conducted about parent-adolescent communication about SRH issues and its determinant factors in Ethiopia were searched. Candidate studies reported in English language were identified through an online search of PubMed, EMBASE, HINARI, Google Scholar, and University repositories. Article search was conducted from May 20 to June 9, 2020. Different search strategies were used to exhaustively search studies to be included in this systematic review and meta-analysis. The search terms used for PubMed data base were: (“adolescent”[MeSH Terms] OR “adolescent”[All Fields]) OR “YOUNG CHILD” [All Fields]) AND (“parents”[MeSH Terms] OR “parents”[All Fields] OR “parent”[All Fields]) OR “guardian” [MeSH terms] OR guardians [MeSH terms] AND (“communication”[MeSH Terms] OR “communication”[All Fields]) AND (“sexual behavior”[MeSH Terms] OR (“sexual”[All Fields] AND “behavior”[All Fields]) OR “sexual behavior”[All Fields] OR “sexual”[All Fields]) AND (“reproductive health”[MeSH Terms] OR (“reproductive”[All Fields] AND “health”[All Fields]) OR “reproductive health”[All Fields]) AND “issues”[All Fields] AND (“ethiopia”[MeSH Terms] OR “ethiopia”[All Fields]).

**Fig. 1:**
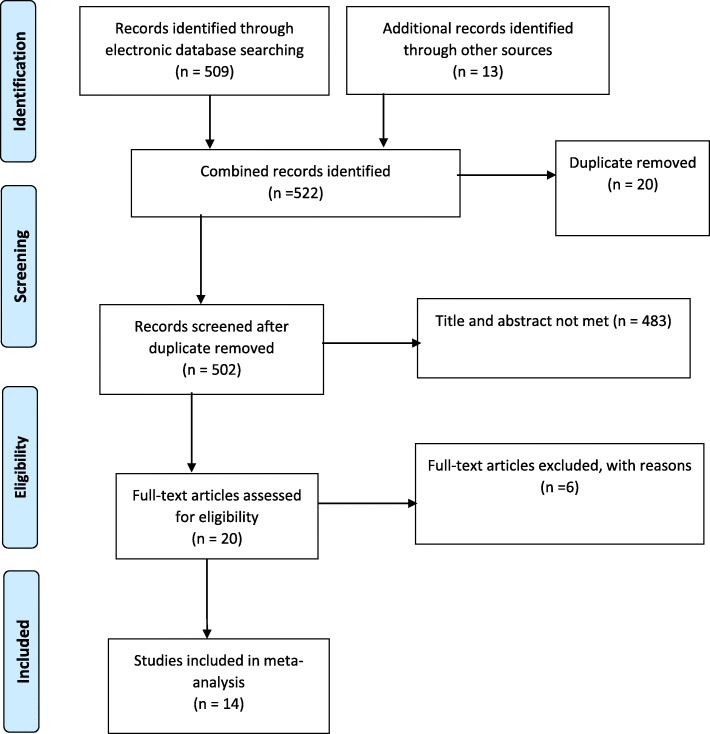
PRISMA flow chart revealing study selection for systematic review and meta-analysis of parent-adolescent communication in Ethiopia

Similarly, a separate search terms were used to search candidate studies for the systematic review and meta-analysis using Google scholar data base. The search terms used to search in Google Scholar were “Adolescent” OR “young child” AND “parent” OR “parents” OR “guardian” OR “guardians” AND “communication” OR “discussions” AND “sexual behaviors” or “sexual and reproductive health issues” AND “associated factors” AND “Ethiopia”. Selection and exclusion of studies for the systematic review and meta-analysis was detailed using the Preferred Reporting Items for Systematic Reviews and Meta-Analyses (PRISMA) guidelines (Fig. 1).

### Inclusion and exclusion criteria

Published and unpublished studies which reported magnitude of parent-adolescent communication on SRH and its determinants factors published/conducted from January 1, 2000 to June 9, 2020 were included.

### Study area

Studies conducted in different Regions of Ethiopia were included in this systematic review and meta-analysis.

### Study design

All studies included in this systematic review and meta-analysis were cross-sectional studies reporting findings from primary data.

### Language

Studies reported in English language were included.

### Population

Studies conducted among adolescent population about parental communication on SRH issues were included.

### Publication condition

Peer reviewed published and unpublished studies were included for the systematic review and meta-analysis.

### Exclusion criteria

Studies with no accessible full text, not reporting outcome of interest, and studies with different target population of our interest were excluded.

### Data abstraction

Data abstraction was conducted using a format prepared in Microsoft excel. The data abstraction format was prepared by authors MM and DA for assessing parent-adolescent communication on SRH issues and its determinant factors. The abstraction format prepared on excel work book includes author name, publication year, Study Region, Study Zone, Study site, study design, study period, sample size, prevalence, and standard error of the prevalence. Similarly, the abstraction form includes factors affecting adolescent parent communication on SRH issues such as knowledge on SRH issues, believe on the importance of discussion on SRH issues, having sexual exposure, parents’ openness to discuss SRH issues, and sex of the participants which could affect parent-adolescent communication on SRH issues.

### Measurement

Parent-adolescent communication regarding sexual issues was the outcome of interest in this systematic review and meta-analysis. Parent-adolescent communication is considered when adolescents communicate at least two SRH issues with their parents**.**

### Data quality assurance

Four reviewers, MM, DA, AM, and WT independently reviewed titles and abstracts of studies to be included in the systematic review and meta-analysis. Then studies were exported to Endnote 7 to manage duplications. Disagreements on inclusion and exclusion of studies were resolved through discussion between authors. Critical appraisal of studies was conducted using Newcastle-Ottawa Quality Assessment Scale (NOS). Studies with quality scores of ≥7 out of 10 scale were considered as low risk for bias and included in the systematic review and meta-analysis (Table 1).

**Table 1:** Characteristics of included studies reporting prevalence of parent-adolescent communication on sexual and reproductive health issues in Ethiopia

Author	Publication year	Region	Study area	Study design	Sample size	Case	Prevalence	Response rate	Risk of bias
Shiferaw et al. [18]	2014	Amhara	Debre Markos	Cross-sectional	697	300	43.042	98.99	Low risk
Ayehu et al. [19]	2016	Amhara	Awabel Woreda	Cross-sectional	781	198	25.35	95.5	Low risk
Fanta et al. [15]	2016	SNNPR	Boditi	Cross-sectional	788	321	40.74	95.8	Low risk
Kusheta et al. [20]	2019	SNNPR	Hadiya Zone	Cross-sectional	428	144	33.64	96	Low risk
Fikre, Martha [21]	2008	SNNPR	Hawassa	Cross-sectional	694	205	29.54	97.1	Low risk
Feyisa, Mulugeta	2017	Oromiya	Fiche	Cross-sectional	394	118	29.95	96	Low risk
Tsegay, Mulugeta [22]	2014	SNNPR	Wolayita Sodo University	Cross-sectional	844	717	84.95	96.6	Low risk
Mekonen et al. [14]	2018	Amhara	Woldiya	Cross-sectional	693	205	29.58	97.3	Low risk
Mekie et al. [12]	2019	Amhara	Debre Tabor	Cross-sectional	394	270	68.53	100	Low risk
Beyene Demissew [23]	2015	Oromiya	Ambo	Cross-sectional	639	540	84.51	98.6	Low risk
Yowhanes et al. [24]	2013	Tigray	Mekelle	Cross-sectional	521	300	57.58	97	Low risk
Tadele et al. [25]	2018	Amhara	Debre Markos	Cross-sectional	394	114	28.93	100	Low risk
Zewudu, Solomon [26]	2006	Addis Ababa	Addis Ababa	Cross-sectional	357	119	33.33	96.2	Low risk
Habte et al. [29]	2019	Oromiya	Robe	Cross-sectional	394	168	42.64	100	Low risk

### Statistical analysis

Microsoft excel was used for data extraction and analysis was performed using Stata version 11 software. Funnel plot and Egger’s regression asymmetry test were used to assess publication bias. On the other hand, the prevalence and factors associated with parent-adolescent communication on SRH issues were presented using forest plots with 95% confidence interval. Moreover, Cochran (Q test) and I^2^ test were used to assess the random variations between primary studies [27]. In this systematic review and meta-analysis, heterogeneity was interpreted as an I^2^ value = 0% as no heterogeneity, 25% = low heterogeneity, 50% = moderate heterogeneity, and 75% = high heterogeneity [28]. To estimate the pooled prevalence of parent-adolescent communication on SRH issues and its associated factors, we have used both fixed and random models for studies with no heterogeneity and with heterogeneity respectively.

## Results

A total of 522 studies (509 and 13) were retrieved through electronic data bases and other sources including university repositories. Twenty two studies were excluded due to duplicates, giving 502 studies. Following reviews of tittles and abstracts, 483 studies were excluded. Twenty full articles were screened for inclusion and six were excluded due to different reasons. Finally, 14 studies were selected for the systematic review and meta-analysis.

### Characteristics of included studies

In this review, a total of 14 studies which reported parent-adolescent communication on SRH issues and determinant factors were included in the analysis. A total of 8018 adolescents were included in this review to estimate the pooled prevalence of adolescent parent communication in Ethiopia. Studies published from January 2000 to June 2020 were included in the systematic review and meta-analysis. The included studies were conducted in 5 Regions of Ethiopia; Amhara Region [12, 14, 18, 19, 25], Oromiya Region ([23, 29]; Feyisa M: Parent-adolescent Sexual and Reproductive Health Communication and Associated Factors Among Secondary And Preparatory School Students In Fiche Town, North Shoa, Oromia Regional State, unpublished), Southern Nations Nationalities and People Region (SNNPR) [15, 20–22], Tigray Region [24] and the capital Addis Ababa [26] see detail (Table 1).

### Meta-analysis

#### Prevalence of parent-adolescent communication in Ethiopia

**Fig. 2:**
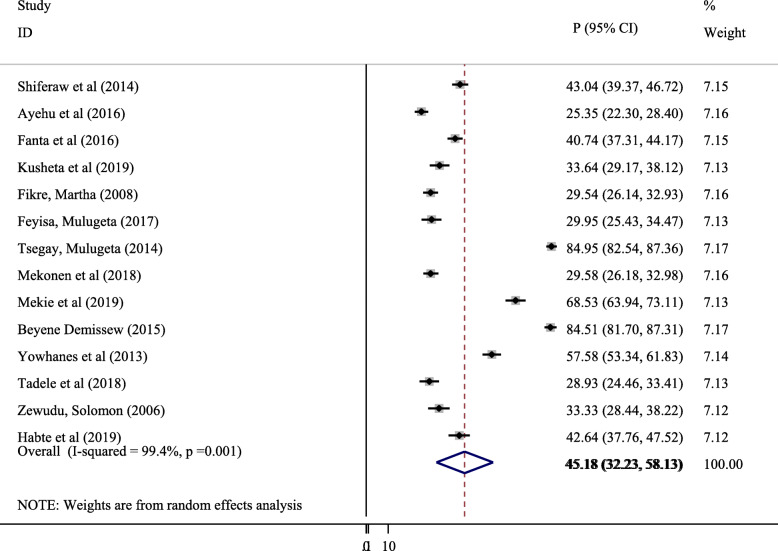
Forest plot of the pooled prevalence of parent-adolescent communication on SRH issues in Ethiopia

Fourteen studies were included to estimate the pooled prevalence of parent-adolescent communication on SRH issues ([12, 14, 15, 18–26, 29]; Feyisa M: Parent-adolescent Sexual and Reproductive Health Communication and Associated Factors Among Secondary And Preparatory School Students In Fiche Town, North Shoa, Oromia Regional State, unpublished). In this regard, the pooled prevalence of parent-adolescent communication on SRH issues in Ethiopia was found to be 45.18% (95%, CI, 32.23, 58.13%) (Fig. 2).

#### Heterogeneity assessment of included studies

**Fig. 3:**
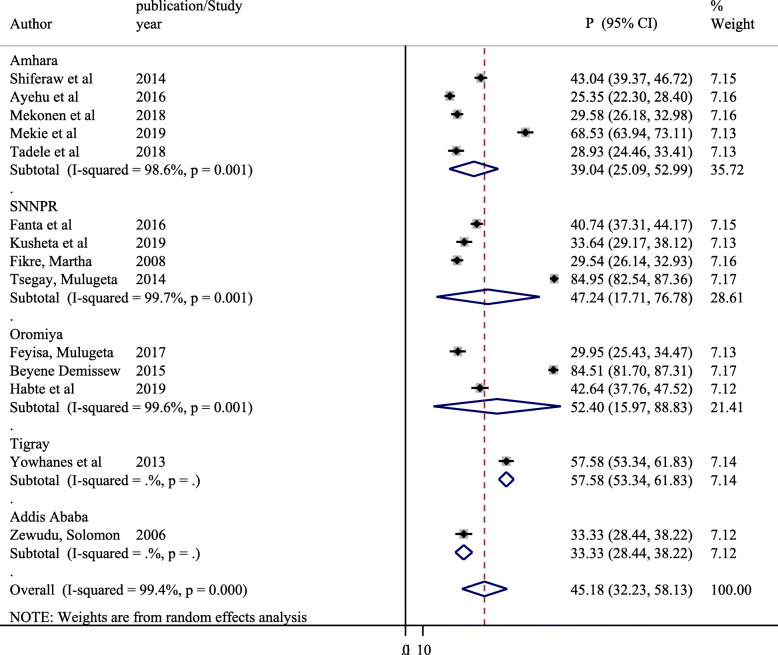
Subgroup analysis of pooled prevalence of parent-adolescent communications on SRH issues based on study Region

**Fig. 4:**
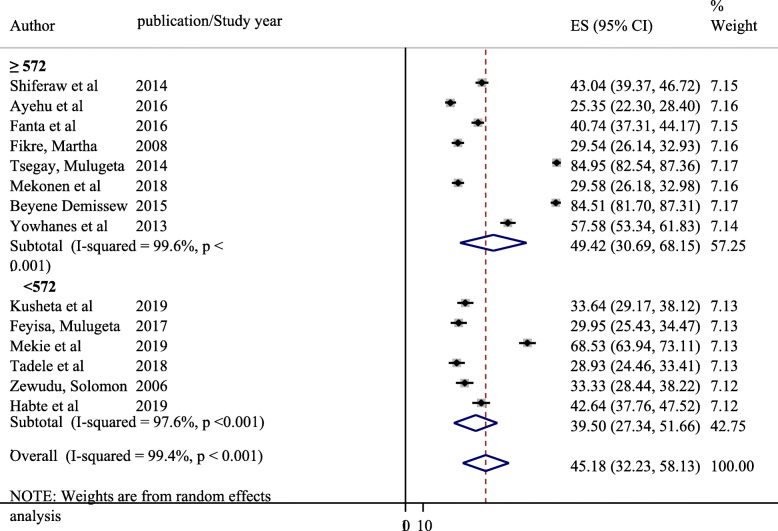
Subgroup analysis of pooled prevalence of parent-adolescent communications on SRH issues based on study Region

A significant heterogeneity was observed between the included primary studies with I squared =99.4%, *P* = 0.001 in computing the pooled prevalence of parent-adolescent communication on SRH issues. Hence, subgroup analysis was performed based on study Regions (Fig. 3) and sample size (Fig. 4).

#### Sub group analysis of parent-adolescent communication on SRH issues based on study Region

The subgroup analysis of the prevalence of parent-adolescent communication on SRH issues based on study Region indicated that there was variations in the prevalence of parent-adolescent communications between Regions with significant heterogeneity within studies (Fig. 3). The highest prevalence of parent-adolescent communication on SRH issues was reported to be the highest in Oromiya Region 52.40% (95%, CI: 15.97, 88.83%) followed by SNNPR, and Amhara Region with respective prevalence of 47.24% (95%, CI: 17.71, 76.78%) and 39.04% (95%, CI, 25.09, 52.99%). However, a wide confidence intervals were found among studies in Oromiya Region and SNNPR. On the other hand, since the included studies in the meta-analysis were a few in Addis Ababa and Tigray, the subgroup analysis findings were not reported in comparison with other Regions.

#### Subgroup analysis of prevalence of parent-adolescent communication on SRH issues based on sample size

Subgroup analysis of prevalence of parent-adolescent communication on SRH issues based on sample size was performed by taking the mean sample size of the included studies as ≥ 527 and < 572 (Fig. 4). The subgroup analysis based on sample size indicated that the prevalence of parent-adolescent communication was found to be higher among studies with sample size of ≥ 527 (49.42, 95% CI: 30.69, 68.15%) compared to studies with sample size of < 527 (39.50, 95% CI: 27.34, 51.66%,) with a significant heterogeneity between studies in both groups.

#### Publication bias

**Fig. 5:**
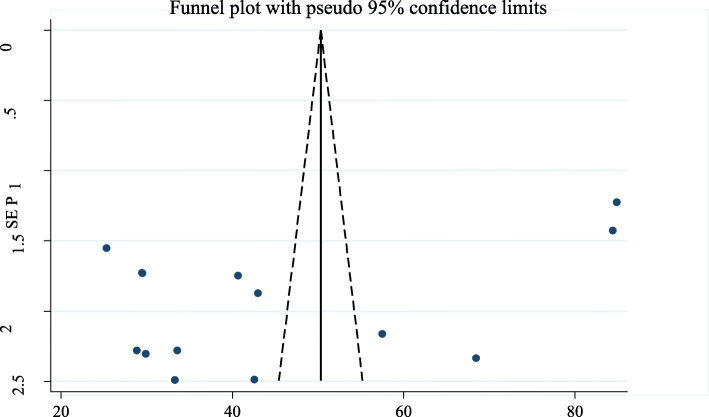
Funnel plot for assessing publication bias of the prevalence of parent-adolescent communication on SRH issues in Ethiopia

Funnel plot and Egger's asymmetry test were used to assess the symmetry of studies used to estimate the pooled prevalence of parent-adolescent communication on SRH issues (Fig. 5). The Egger’s regression asymmetry test indicated that there was a minimal evidence of publication bias with *p* value of 0.044.

### Factors associated with parent-adolescent communication in Ethiopia

In this systematic review and meta-analysis, knowledge on reproductive health matters (OR = 2.91, 95% CI:1.21, 7.01), believe on the importance of discussion (OR = 4.18, 95% CI: 2.63, 6.65), history of sexual intercourse (OR = 1.95, 95% CI: 1.53, 2.50), parents’ openness (OR = 3.39, 95% CI: (2.48, 4.62), and being female adolescent (OR = 1.60, 95% CI:1.07, 2.38) were significant predictors of parent-adolescent communications on SRH issues.

#### The association between knowledge on reproductive health matters and parent-adolescent communication on SRH issues

**Fig. 6:**
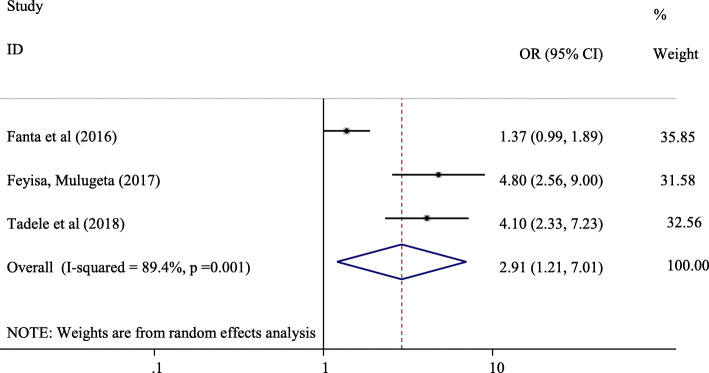
Forest plot of the association between knowledge of reproductive health issues and parent-adolescent communication in Ethiopia

Three studies ([15, 25]; Feyisa M: Parent-adolescent Sexual and Reproductive Health Communication and Associated Factors Among Secondary And Preparatory School Students In Fiche Town, North Shoa, Oromia Regional State, unpublished) were incorporated in the analysis of the association between adolescents’ knowledge on reproductive health matters and adolescent parent communication on SRH issues. Adolescents who have knowledge about reproductive health matters were 2.91 times more likely to communicate SRH issues with their parents compared with those who did not have knowledge (OR = 2.91, 95% CI:1.21, 7.01) (Fig. 6).

#### Believe on the importance of discussion on reproductive health matters and its association with communication on SRH issues

**Fig. 7:**
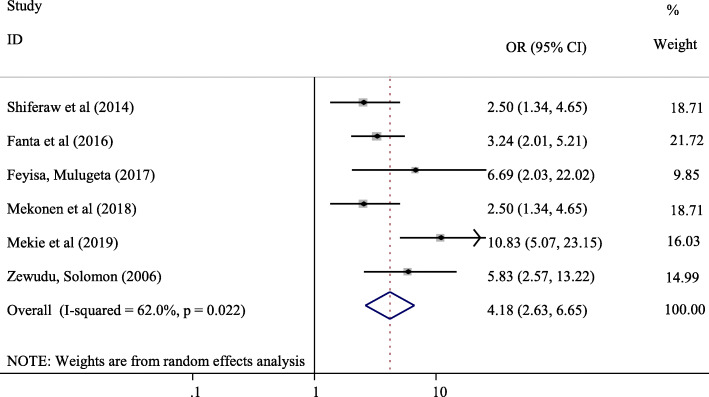
Forest plot of the association between beleif on the importance of discussion on SRH issues and parent-adolescent communication in Ethiopia

The finding of our meta-analysis indicated that there was a statistically significant association between believe on the importance of discussion on reproductive health matters and parent-adolescent communications on SRH issues. Adolescents who believed on the importance of discussion on reproductive health matters were 4.18 times more likely to communicate SRH issues with their parents compared with those who did not believe on the importance of discussing sexual health (OR = 4.18, 95% CI: 2.63, 6.65). Six studies ([12, 14, 15, 18, 26]; Feyisa M: Parent-adolescent Sexual and Reproductive Health Communication and Associated Factors Among Secondary And Preparatory School Students In Fiche Town, North Shoa, Oromia Regional State, unpublished) were included in the meta-analysis of the association between believe on the importance of discussion on reproductive health matters and parent-adolescent communications on SRH issues (Fig. 7).

#### The association between sexual exposure and parent-adolescent communication on SRH issues

**Fig. 8:**
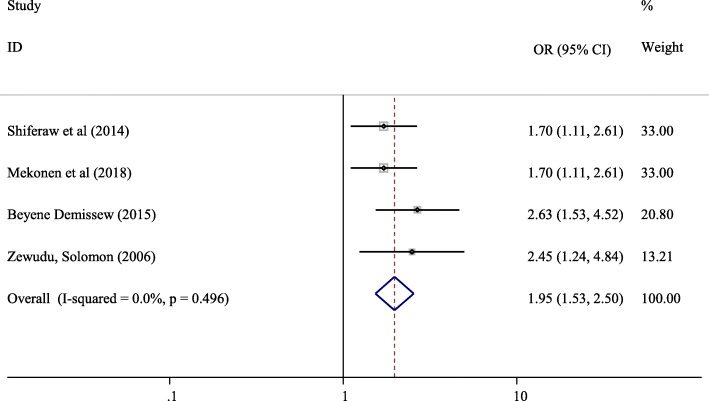
Forest plot of the association between history of sexual intercourse and parent-adolescent communication on SRH issues in Ethiopia

Four studies were included in the meta-analysis of the association between history of sexual intercourse and parent-adolescent communication on SRH issues [14, 18, 23, 26]. Adolescents who had history of sexual exposure were 1.95 times more likely to communicate on SRH issues with their parents compared with adolescents who did not have sexual exposure (OR = 1.95, 95% CI: 1.53, 2.50) (Fig. 8).

#### The association between parents’ openness and parent-adolescent communication on SRH issues in Ethiopia

**Fig. 9:**
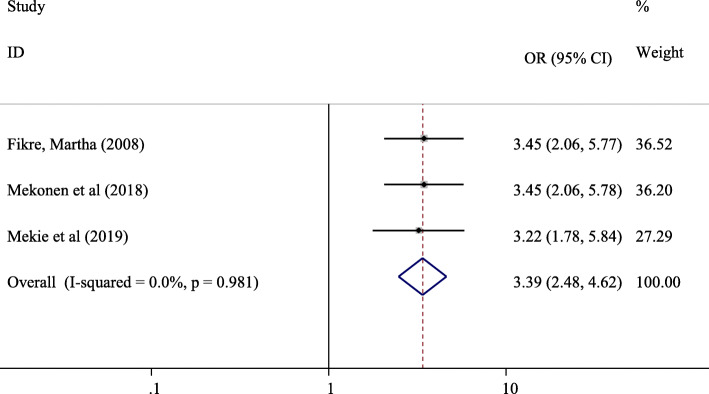
Forest plot showing the association between parents’ oppenness and parent-adolescent communication on SRH issues

Three studies [12, 14, 21] were included in the analysis of parent-adolescent communication on SRH issues (Fig. 9). The odds of experiencing parent-adolescent communication on SRH issues were 3.39 times higher among participants whose parents were open enough to discuss SRH issues compared with counterparts (OR = 3.39, 95% CI: (2.48, 4.62).

#### Association between sex of the study participants and parent-adolescent communication on SRH issues

**Fig. 10:**
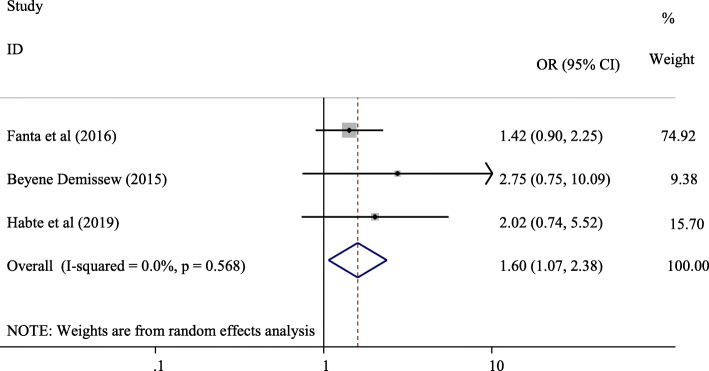
Forest plot of the association between female sex and pareant-adolescent communication on SRH issues

Three studies [15, 23, 29] were used to analyze the association between sex of the study participants and parent-adolescent communication on SRH issues (Fig. 10). The odds of having parent-adolescent communication on SRH issues were 1.60 times higher among female participants compared with males (OR = 1.60, 95% CI:1.07, 2.38).

## Discussion

Parent-adolescent communication promotes adolescents’ self-esteem and boosting their confidence to prevent risky sexual behaviors since parents are the primary sexual educators for young child. Hence, encouraging adolescents to talk to their parents about sensitive issues is imperative [30]. This systematic review and meta-analysis was intended to examine the pooled prevalence of parent-adolescent communication on SRH issues and its determinant factors in Ethiopia. The pooled prevalence of parent-adolescent communication on SRH issues in Ethiopia was found to be 45.18% (95%, CI: 32.23, 58.13%). The finding of our meta-analysis is found to be higher than a study conducted in Nigeria which reported 37.4, 32.5 and 9.5% parent-adolescent communication about HIV/AIDS, family planning, and contraception respectively [31]. In a similar manner, the level of parent-adolescent communication on SRH issues in our study was found to be higher than a study conducted in Rwanda which reported no parent-adolescent discussion among 81% of the study participants [32]. The difference might be attributed to variation in measurement of the outcome variable and sociocultural condition of the society.

Adolescents who did have knowledge about reproductive health matters were 2.91 times likely to communicate SRH issues with their parents compared with those who did not have knowledge about reproductive health issues (OR = 2.91, 95% CI:1.21, 7.01). Knowledge on reproductive health matters was also reported to be statistically significant with parent-adolescent communications on SRH issues in an intervention study by Ford et al. The study indicated that adolescents in the sexual health intervention group reported a higher mean frequency score for parent-adolescent communication about sex compared with control counterparts [33]. Similarly, believe on the importance of discussion on SRH issues was found to be the predictor of parent-adolescent communication on SRH issues in in this systematic review and meta-analysis. Adolescents who believed on the importance of discussion on reproductive health matters were 4.18 times more likely to communicate SRH issues with their parents compared with those who did not believe on the importance of discussing sexual health (OR = 4.18, 95% CI: 2.63, 6.65). Other studies indicated that parents are the initiators of parent-adolescent communications which indicates adolescents’ low belief on the importance of parent-adolescent communication on SRH issues [30, 34]. Hence, evidence based education shall be provided to adolescents about the significance of parent-adolescent communication on SRH issues to improve parent-adolescent communication which in turn reduce adolescents’ engagement in risky sexual behaviors.

Our systematic review and meta-analysis indicated that Adolescents who had history of sexual exposure were 1.95 times more likely to communicate on SRH issues with their parents compared with adolescents who did not have sexual exposure (OR = 1.95, 95% CI: 1.53, 2.50). The finding of our study is supported by a study conducted in United States of America which reported 2.0 times higher odds of parent-adolescent communication on SRH issues among participants who ever had sexual intercourse compared with counterparts [35].

The odds of practicing parent-adolescent communication on SRH issues were found to be 3.39 times higher among participants whose parents were open enough to discuss SRH issues compared with counterparts (OR = 3.39, 95% CI: 2.48, 4.62). The finding of this systematic review and meta-analysis is supported by a study conducted in Johannesburg, South Africa which reported non-conducive environment for open discussions of SRH issues was a barrier for parent-adolescent communication on sexual issues [34]. Parents might not be open for their child in regards to sexual matters due to cultural belief that talking sexual matters with adolescents might facilitate sexual initiation and experimentations among adolescents. This belief need to be tackled by providing evidence based education for target audiences. Interventions targeting both adolescents and their parents may increase effectiveness in reducing negative adolescent sexual and reproductive health outcomes [36, 37].

This systematic review and meta-analysis found that sex of the study participant was a determinant factor for parent-adolescent communication on SRH issues. The odds of having parent-adolescent communication on SRH issues were 1.60 times higher among female participants compared with males (OR = 1.60, 95% CI:1.07, 2.38). The finding of our systematic review and meta-analysis is supported by studies conducted in Nairobi, Kenya [38], Uganda [39], and Nigeria [31] which reported boys as less likely to discuss SRH issues with their parents than girls. In the same manner, a similar finding was reported in a meta-analysis by Widman et al. which reported a more pronounced protective role of parent communication among girls than boys with regards to safer sex behavior [40].

### Limitations of the study

Despite it was intended to assess the magnitude of parent-adolescent communications on SRH issues in Ethiopia, we could not get studies from all Regions of the country which might affect its representativeness. Small numbers of studies were used to analyze the pooled effect of some variables due to shortage of studies in the country. However, we have used both published and unpublished studies which might reduce the risk of publication bias.

## Conclusion

The prevalence of parent-adolescent communication on SRH issues was found to be low. Knowledge of adolescents about reproductive health matters, believe on the importance of discussion on reproductive health issues, history of sexual exposure, parents’ openness to discuss SRH issues, and being female were found to be the positive predictors of parent-adolescent communication on SRH issues in Ethiopia. The finding of this systematic review and meta-analysis indicated that education about reproductive health matters is significant to improve adolescent parent communication on SRH issues which in turn reduces reproductive health problems among adolescent population. Incorporation of reproductive health courses in secondary school curriculum would be the best option.

## Data Availability

All relevant data are presented within the manuscript and the dataset used to reach conclusion can be assessable from the corresponding author on request.

## Abbreviations

AIDS: Acquired Immuno-Deficiency Syndrome
HIV: Human Immuno-Deficiency Virus
NOS: Newcastle-Ottawa Quality Assessment Scale
SRH: Sexual and Reproductive Health
SNNPR: Southern Nations Nationalities and People Region
WHO: World Health Organization
